# Utilization of APPswe/PS1dE9 Transgenic Mice in Research of Alzheimer's Disease: Focus on Gene Therapy and Cell-Based Therapy Applications

**DOI:** 10.4061/2011/517160

**Published:** 2011-10-30

**Authors:** Tarja Malm, Jari Koistinaho, Katja Kanninen

**Affiliations:** ^1^Department of Neurobiology, A.I. Virtanen Institute for Molecular Sciences, University of Eastern Finland, P.O. Box 1627, 70211 Kuopio, Finland; ^2^Department of Oncology, Kuopio University Hospital, P.O. Box 1777, 70211 Kuopio, Finland; ^3^Department of Pathology, The University of Melbourne, Melbourne, VIC 3010, Australia

## Abstract

One of the most extensively used transgenic mouse model of Alzheimer's disease (AD) is APPswe/PS1dE9 mice, which over express the Swedish mutation of APP together with PS1 deleted in exon 9. These mice show increase in parenchymal A**β** load with A**β** plaques starting from the age of four months, glial activation, and deficits in cognitive functions at the age of 6 months demonstrated by radial arm water maze and 12-13 months seen with Morris Water Maze test. As gene transfer technology allows the delivery of DNA into target cells to achieve the expression of a protective or therapeutic protein, and stem cell transplantation may create an environment supporting neuronal functions and clearing A**β** plaques, these therapeutic approaches alone or in combination represent potential therapeutic strategies that need to be tested in relevant animal models before testing in clinics. Here we review the current utilization of APPswe/PS1dE9 mice in testing gene transfer and cell transplantation aimed at improving the protection of the neurons against A**β** toxicity and also reducing the brain levels of A**β**. Both gene therapy and cell based therapy may be feasible therapeutic approaches for human AD.

## 1. Introduction

Recent advances in the field of gene transfer technology have allowed the delivery of DNA into target cells of the recipient based on the use of viral systems for gene therapy. This technology allows the delivery of DNA to target cells to achieve the expression of a protective or therapeutic protein and also in neurodegenerative diseases, including Alzheimer's disease (AD). On the other hand, the cell therapy-based applications in combating AD are based on the rationale of replacement of functionally lost neurons by transplantation of neuronal stem or progenitor cells, improvement in diminished neuronal function by creating an environment aiding at neuronal recovery, or clearing of toxic beta-amyloid (A*β*) plaques by phagocytic cells. Since AD is a multifactorial disorder that progresses slowly, it is important to choose a gene transfer approach that allows lengthy expression of the therapeutic gene and/or a cell-based therapeutic strategy that results in sufficient and preferably long-term reduction in A*β* levels. The central nervous system (CNS) is a unique site that poses challenges to the delivery of therapeutics as efficient delivery requires the crossing of the blood brain barrier (BBB). The targets of gene therapy for AD fall into four main categories: catabolism of amyloid precursor protein (APP) and removal of A*β*, neuroprotective genes, growth factors, and apolipoprotein E (ApoE) alleles. The main strategies adopted for viral vector-mediated gene delivery to the CNS include direct delivery into the brain parenchyma and peripheral delivery. Similarly, although the brain is an evident target tissue of the cell-based therapy, cells administered peripherally may function also in peripheral sites. APPswe/PS1dE9 mice accumulate toxic A*β* in the brain parenchyma, and also around the blood vessels as cerebral amyloid angiopathy (CAA) upon aging, making them excellent models for AD's amyloidosis. Importantly, similar to some clinical AD cases, the behavioral deficits correlate with the soluble A*β* levels in the brain of this mouse line. Therefore, these mice offer a valuable tool in studies of cell mediated A*β* clearance. Studies utilizing APPswe/PS1dE9 mice as a model of AD are discussed below in the context of virus vector-mediated and cell-based experimental therapy* in vivo.*

## 2. APPswe/PS1dE9 Mice as a Model of AD

AD is a multifactorial disorder leading to progressive memory loss and eventually death. One of the pathological features of the disease is the abnormal accumulation of toxic A*β* peptides in the brain parenchyma [1, 2]. These peptides are cleavage products derived from the amyloid precursor protein (APP) through endoproteolytic cleavage operated by specific secretases, BACE-1 and *γ*-secretase [1, 2]. APP mutations alter the processing of the protein by shifting the nonamyloidogenic processing towards amyloidogenic processing which eventually leads to generation of highly fibrillogenic, toxic A*β*1-42 peptides [1, 2]. Presenilin-1 (PS-1) and presenilin-2 (PS-2) function as a catalytic site for *γ*-secretase and mutations in PS-1 or PS-2 further increase the production of amyloidogenic A*β* [1, 2]. Human AD neurons also contain intraneuronal inclusions of hyperphosphorylated tau protein, called neurofibrillary tangles (reviewed by [3, 4]). These abnormal protein inclusions alter neuronal function and result in neuron death. Mutations in APP and PS1 have been linked to familial, inherited forms of AD, which account less than 10 % of the clinical AD cases (reviewed by [5, 6]). Indeed, the majority of the diagnosed AD patients have a sporadic form of the disease in which the underlying cause remains unknown. Mutations in tau have not been linked to clinical AD but are the underlying cause of another neurodegenerative disorder called frontotemporal dementia [3, 4]. 

AD has been widely studied exploiting various *in vitro* models and with the development of molecular biology methods, transgenic mouse models have become increasingly popular. Up to date, a wide range of transgenic mouse models based on APP expression have been generated (reviewed by [7, 8]). Despite their utmost importance in the field of AD research, none of the models developed so far are able to recapitulate the full neuropathological features of clinical human AD. In general, APP transgenic mice develop A*β* plaques and memory deficits but lack frank neuron loss and neurofibrillary tangles [7, 8]. Moreover, not all APP transgenics develop memory deficits independent of A*β* aggregation and not all plaque bearing mice develop memory deficits. Overexpression of the PS1 gene alone does not result in plaques, but together with APP, it hastens the development of plaques; tau overexpression leads to memory deficits and tangle formation but does not result in plaques [7, 8]. Overexpression of all these genes within one mouse line—named the triple transgenic mice—recapitulate memory deficits, plaques, and tangles. 

To date, there is no feasible model for the sporadic form of AD. Some larger animals such as monkeys [9], old dogs [10, 11] and cats [12] develop spontaneous A*β* plaques. AD pathology often coexists with infarcts, *α*-synuclein, and aggregates of TAR DNA-binding protein-43 (TDP-43) [13, 14]. Thus, any model for AD is a compromise and should be chosen to answer the specific question addressed by the study. Nonetheless, with the exception of the time of onset of the disease, pathological features of both sporadic and familial human AD are similar [15]. 

APPswe/PS1dE9 mice, described by Jankowsky et al. in 2004, overexpress the Swedish mutation of APP, together with PS1 deleted in exon 9 [16]. Overexpression of the transgene construct leads to overproduction of APP and PS1 splice variants with concomitant increase in parenchymal A*β* load. These mice develop the first A*β* plaques at 4 months of age. Activated microglia and astrocytes surround the ever growing deposits. By 12 months of age, the mice develop deficits in a widely used behavioral test measuring spatial navigation and reference learning, Morris water maze (MWM), but memory deficits can be seen in radial arm water maze even at 6 months of age [17]. Even though these mice do not exhibit frank neuronal loss, the APPswe/PS1dE9 mice display a variety of other clinically relevant AD-like symptoms. These include mild neuritic abnormalities [18], local plaque-related loss in neuronal activity [19], increased mortality, high prevalence to unprovoked seizures [20], and age-dependent deficits in the pre- and postsynaptic cholinergic transmission [21] and similar to some clinical AD cases, the soluble A*β* levels correlate with behavioral deficits in these mice at 12 months of age [22]. Due to the fact that the mice develop parenchymal A*β* pathology and memory deficits evidenced already by 6 months of age [17], these mice offer a valuable tool in studies aiming at the development of new therapeutic approaches targeted specifically against the plaques and related neuroinflammation.

## 3. Gene Transfer-Based Therapy Applications

Recent advances in the field of gene transfer technology have allowed the delivery of DNA into target cells of the recipient based on the use of viral systems for gene therapy. This technology allows the delivery of DNA to target cells to achieve the expression of a protective or therapeutic protein. Viral vectors are a powerful tool that allows transgene delivery to specific locations *in vivo*, yet numerous aspects must be considered in order to achieve efficient gene delivery. These include choice of viral vector, mode and location of delivery, duration and location of transgene expression, and potential toxicity associated with the approach. Delivery issues and immune responses to viral vectors remain the major limitations of CNS gene transfer (reviewed in [23, 24]).

The CNS is a unique site that poses challenges to the delivery of therapeutics as efficient delivery requires the crossing of the BBB. While there are a large number of techniques to transfer genes *in vivo*, the main strategies adopted for viral vector-mediated gene delivery to the CNS include direct delivery into the brain parenchyma and peripheral delivery. Direct delivery into the brain allows targeted expression of the gene of interest in a relatively limited area. The stereotactic surgical procedure is invasive but relatively safe as the immune response is minimal when the vector is injected directly into the CNS. Moreover, transduction can be performed unilaterally, thus allowing an opportunity to compare alterations in the contralateral side of the same animal. Peripheral delivery relies on the ability of viral vectors to undergo transport to other regions of the CNS. Viral vectors can be targeted to multiple regions via minimally invasive administration, including intravenous administration and intramuscular injections.

Several diseases of the CNS require long-term treatment. Since AD is a multifactorial disorder that progresses slowly, it is important to choose an approach that allows lengthy expression of the therapeutic gene. Lentivirus (LV), adenovirus (AV), adeno-associated virus (AAV) and baculovirus- (BV-) derived gene transfer vehicles have been produced, each with advantages and potential drawbacks. LV vectors are emerging as one of the preferred gene delivery candidates for CNS because of their ability to transfer a relatively large transgene into nondividing neuronal cells. While LV integration into the host genome allows long-term transgene expression, there is a low risk of insertional mutagenesis (reviewed in [25]). AV vectors can also infect most cell types, including neurons and can be manipulated to accommodate relatively large DNA inserts. However, they have potential for therapies of limited duration, as permanent expression of the transgene is not achieved. While AAV vectors are in many ways comparable to LV vectors, their major drawback is that their cloning capacity is relatively small, precluding the transfer of genes over five kilobases in size (reviewed in [26]). BV represents still another interesting viral vector with some advantages as a gene therapy vector [27]. BVs can be easily and quickly produced in high titers, and they can transduce both dividing and G1/S-arrested cells. They are also relatively safe, because insect host-derived viruses do not replicate in vertebrate cells. However, because the virus is produced in insect cells, which results in the display of foreign glycoproteins, the risk of immunogenic responses and inactivation by the blood complement system by classical pathway is high [27]. However, as the brain is an immune privileged tissue, the risks of immunogenic responses remain quite minimal. BVs seem to be especially useful for the targeting of choroid plexus cells.

The targets of gene therapy for AD fall into four main categories: catabolism of APP and removal of A*β*, neuroprotective genes, growth factors, and ApoE4 alleles (reviewed in [28]). Studies utilizing APPswe/PS1dE9 mice as a model of AD are discussed below in the context of virus vector-mediated experimental *in vivo* gene therapy.

## 4. Therapeutic Approaches Targeted to Virus Vector-Mediated Removal of A*β*

Depletion of A*β* in affected areas of the brain can be achieved by using viral vectors to deliver small interfering RNAs for enzymes involved in APP catabolism, antibodies that reduce the amount of existing A*β*, and enzymes that degrade A*β*. Animal models of AD have been utilized to test the therapeutic and disease-modifying abilities of each of these approaches *in vivo*. The studies reviewed below have utilized the APPswe/PS1dE9 mouse model to experimentally assess the potential of virus vector-mediated gene delivery targeted to A*β* removal.

Delivery of A*β* antibodies is known to reduce A*β* burden in mice modelling AD. In 2009, Wang et al. delivered an anti-A*β* single-chain antibody (scFv) intrahippocampally, intraventracularly or intramuscularly into 9-month old APPswe/PS1dE9 mice [29]. The transgene was robustly expressed for up to three months when an AAV vector was utilized as the gene transfer vehicle. Immunohistochemical analyses showed that the A*β* burden was reduced by approximately 30% in the hippocampi of scFv injected mice. Interestingly, ELISA analyses showed that both intramuscular and intracranial delivery of the gene also clearly reduce A*β* burden. This suggests that targeting the brain metabolites relevant for the disease pathology and leaking out of the brain by peripheral administration of a viral vector is a good alternative to the more invasive intracranial administration. In the brain, transgene expression colocalized with neuronal, but not glial markers, suggesting that the transgene was mainly expressed in neurons. Importantly, gene transfer did not elicit an inflammatory reaction. More recently, the same group reported that intramuscular delivery of the same vector into 3-month-old APPswe/PS1dE9 mice attenuated A*β* accumulation and cognitive impairment when analyzed 6 months later [30]. Intramuscular delivery of the vector resulted in transgene expression at the site of injection, in the liver, and in the olfactory bulb without concomitant inflammation. Peripheral administration of a viral vector carrying scFv is a potentially safe and less invasive alternative to intracranial gene transfer. 

Neprilysin is a major enzyme capable of degrading A*β* in the brain [31, 32]. Reduction of neprilysin occurs in early stages of AD and it is downregulation contributes to A*β* accumulation in the brain [33–37]. To assess the therapeutic potential of neprilysin gene replacement, El-Amouri et al. used an LV vector as a gene delivery system to over-express human neprilysin in 3-month-old APPswe/PS1dE9 mice [38]. The vector was injected into the cortical/hippocampal area and the mice were assessed four months after gene delivery. Using this approach, neprilysin was overexpressed threefold, and the majority of the expression occurred in neurons. The overexpression of neprilysin reduced cognitive impairment and correlated with a reduction in amyloid burden and attenuation of oxidative stress and glial activation. These findings are in line with beneficial effects of virus vector-mediated neprilysin over-expression studies reported in other mouse models of AD [39–43].

## 5. Therapeutic Approaches Targeted to Virus Vector-Mediated Overexpression of Protective Genes

Vast evidence exists for the role of oxidative stress in the pathogenesis of AD (reviewed in [44, 45]). An endogenous defense system is activated during oxidative stress that aims to alleviate the harm caused by reactive oxygen species. Nuclear factor erythroid 2-related factor 2 (Nrf2) is a transcription factor that is activated during oxidative stress to induce defensive gene expression that promotes cell survival (reviewed in [46, 47]). The Nrf2 pathway is attenuated in human AD brain [48] and concomitantly with increased A*β* deposition in the APPswe/PS1dE9 and other APP/PS1 mouse models [49, 50]. To exploit the Nrf2 pathway therapeutically, we recently overexpressed human Nrf2 in the hippocampi of APPswe/PS1dE9 mice using an LV vector [51]. At the time of gene therapy, the mice were nine months of age and exhibiting full-blown AD pathology. Assessment of the mice six months after gene transfer revealed robust and sustained gene expression especially in neurons without associated toxicity. Modulation of Nrf2 levels in the brain attenuated memory impairment and reduced astroglial activation, suggesting that LV-mediated gene transfer of an endogenous protective gene is beneficial against oxidative stress associated with AD.

## 6. APPswe/PS1dE9 Mice in the Development of Cell Therapy

The cell therapy-based applications in combating AD are based on the rationale of replacement of functionally lost neurons by (i) transplantation of neuronal stem or progenitor cells, (ii) improvement in diminished neuronal function by creation of an environment aiding at neuronal recovery, or (iii) clearing of preexisting toxic A*β* plaques by phagocytic cells. 

Even though loss of cholinergic neurons is an evident part of AD pathology, APPswe/PS1dE9 mice, similar to most other AD transgenic mouse models, do not exhibit frank neuronal loss [16, 18]. In fact, only the triple transgenic mice show some degree of neuronal loss [52]. The APPswe/PS1dE9 mice have recently been shown to have reduced release of acetylcholine and decreased potency of carbachol-induced G-protein activation in cortical membranes, indicating that mild deficits do occur in the cholinergic system without clear neuronal loss [18, 53]. On the other hand, APPswe/PS1dE9 mice accumulate toxic A*β* in the brain parenchyma, and also around the blood vessels, as CAA upon aging, making them excellent models for Alzheimer's amyloidosis [54]. Importantly, similar to some clinical AD cases, the behavioral deficits correlate with the soluble A*β* levels in the mouse brain [55]. Therefore, these mice offer a valuable tool in studies of cell-mediated A*β* clearance. Indeed, all cell-based therapy applications developed using the APPswe/PS1dE9 mice aim primarily to remove toxic A*β* peptides. Cell transplantation studies using other than phagocytic cell types, such as neuronal progenitor cells, have utilized different transgenic mouse models (APP23 mice, [56]). 

## 7. Bone Marrow Stem Cell-Based Therapy Approaches

APPswe/PS1dE9 mice were utilized to discover the initial role and contribution of peripheral phagocytic cells in A*β* clearance. One of the primary findings was the contribution of the hematopoietic system to the inflammatory reactions in the AD-like mouse brain. By using novel, bone marrow (BM) chimeric mice (described by [57]), BM-derived monocytic cells were shown to infiltrate into the brains of the APPswe/PS1dE9 transgenic mice [58]. These cells clearly associated with A*β* deposits, suggesting that aggregated A*β* peptides attracted them. When the brains of the APPswe/PS1dE9 mice were challenged with additional inflammatory stimuli, lipopolysaccharide (LPS), infiltration of the peripheral cells was dramatically increased. LPS also increased the association of the peripheral monocytic cells with the A*β* deposits and caused concurrent reduction in the brain A*β* burden. This suggests that the peripheral monocytic cells were actively taking part in the clearance of A*β* [58, 59]. The capacity of the cells to clear A*β* was further proven with an *ex vivo* assay, in which BM monocytic cells were cultivated on top of aged APPswe/PS1dE9 mouse brain sections. These brain sections contained A*β* in its natural conformation resembling the *in vivo* situation in the AD mouse brain. Indeed, monocytic cells efficiently cleared A*β ex vivo* [60, 61]. Moreover, when the cells were transplanted intrahippocampally into the brains of the APPswe/PS1dE9 mice, the cells also cleared A*β in vivo* (Magga et al., [61]), further establishing the foundation for justification into development of BM cell-based therapy for AD.

APPswe/PS1dE9 transgenic mice have also been used in other studies assessing the significance of peripheral immune cells in the clearance of A*β*. Keene and coworkers transplanted lethally irradiated APPswe/PS1dE9 mice with BM cells deficient in prostaglandin E2 receptor subtype 2 (EP2) [60]. EP2 is a receptor mediating microglial proinflammatory reactions. Microglia deficient in EP2 were increasingly capable of A*β* phagocytosis and exhibited less paracrine neurotoxicity. Moreover, APPswe/PS1dE9 transgenic mice lacking this receptor subtype showed reduced cerebral A*β* burden and oxidative damage to neurons upon innate immune activation [60]. Keene et al. demonstrated that deletion of EP2 specifically in peripheral cells was sufficient to enhance their infiltration into the brain to achieve subsequent brain A*β* clearance. The study provides further basis for the development of cell-based therapy for AD. 

An important outcome of potentially efficient therapy applications is the potential improvement of memory deficits. The APPswe/PS1dE9 mice do develop clear memory deficits, which were not assessed in the above-mentioned studies. The fact that the deficits correlate poorly with plaque load and seem to be more dependent on the levels of soluble A*β* [55], raises the question whether the clearance of A*β* by phagocytic cells alone would be sufficient to achieve improvements in memory. This question was addressed in a study by Hao and coworkers, where they transplanted myeloid differentiation factor 88- (MyD 88-) deficient BM cells into APPswe/PS1dE9 mice and detected not only a reduction in the brain A*β* load of the mice, but also, importantly, improvement in behavior [62]. MyD88 is one of the key molecules that mediate pathogen recognition signaling in immune cells. Upon binding to the CD14 and Toll-like receptors, microorganisims—and A*β*—mediate their signaling through MyD88, leading to activation of transcription factors nuclear factor kappa-B and AP-1, which upregulate the transcription of several proinflammatory gene products [63]. Genetic deletion of MyD88 in the BM cells leads to increased capacity of BM-derived macrophages to phagocytose A*β* and reduced inflammatory activation in the brain. 

The studies described above have utilized the APPswe/PS1dE9 mice to suggest that BM cell-based therapy may be both a prominent and feasible therapeutic option for AD in clinics. Importantly, several studies utilizing other AD transgenic mouse lines support these findings and also show the phagocytic capacity of BM-derived monocytic cells [64–66]. This strengthens the clinical relevance of the results obtained with the APPswe/PS1dE9 mice. 

## 8. Mesechymal Stem Cells in the Development of Cell-Based Therapy

Cell-based therapy applications have also been tested with other cell types in mice closely resembling APPswe/PS1dE9 mice, namely, the APP/PS1 mice. These mice carry the same APP transgene as the APPswe/PS1dE9 mice. However, instead of the deletion in exon 9 of the PS1 gene, the APP/PS1 mice carry the mutation A264E in the PS1 gene. APP/PS1 mice have very similar parenchymal A*β* pathology and gliosis as the APPswe/PS1dE9 mice and develop behavioral deficits in MWM at approximately the same age [16]. 

The BM harbors several types of progenitor cells. Mesenchymal stem cells are multipotent cells giving rise to a diversity of cell types including osteoblasts, chondrocytes, and adipocytes. Mesenchymal stem cells have been shown to promote brain repair when transplanted into a damaged brain through release of bioactive molecules and modulation of immune responses (reviewed by [67]). When transplanted several times directly into the brains of the APP/PS1 mice as the pathology of the mice progressed, these cells were also shown to reduce A*β* deposition and restore defective microglial function; the brain inflammatory responses diminished, the protein or mRNA expression of A*β* degrading enzymes increased and tau hyperphosphorylation was reduced. The combination of these effects eventually led to improvement in the cognition of the mice [68].

## 9. Astrocytes in the Development of Cell-Based Therapy Applications

BM derived monocytic cells are not only cell type capable of A*β* clearance. Recently astrocytes have been shown to harbor dramatic A*β* clearing capacity [69, 70]. By using a similar *ex vivo* assay as described above, demonstrated that astrocytes clear A*β* in its naturally occurring conformation on brain sections dissected from APPswe/PS1dE9 mice [70]. Whereas actual A*β* laden microglia or BM derived monocytic cells has only been shown in a few studies, astrocytes clearly colocalized with A*β* as detected by confocal microscopy. Moreover, when astrocytes were transplanted intrahippocampally into APPswe/PS1dE9 mouse brain they also endocytosed A*β in vivo* [70, 71]. In addition to prominent endocytosis, extracellular, A*β* clearing proteases neprilysin, angiotensin converting enzyme -1 and endothelin converting enzyme -2 were upregulated in transplanted astrocytes, suggesting a contributory role for these enzymes in human A*β* clearance by astrocytes [71]. This conclusion was also supported by the *ex vivo* assay of A*β* clearance, which demonstrated that inhibition of any of these proteases also reduced the potency of astrocytes to clear A*β* deposits from the A*β*-rich brain sections of aged APPswe/PS1dE9 mice. Even though reductions in A*β* burden were seen up to 8 weeks after astrocyte transplantation, the transplanted astrocytes eventually degenerated and died through apoptosis [71]. It is not yet clear if loss of transplanted, A*β* clearing astrocytes results in the return of A*β* deposits in amounts expected for AD mouse brain. The results suggest that astrocyte transplantation may have potential only for temporary reduction of A*β* in the AD brain. 

## 10. Feasibility of Stem-Cell Therapy in AD

One of the most interesting questions remaining relates to the issue of feasibility of cell-based therapy for AD. How easy would it be to modulate the patient's own peripheral cells? Several papers describe the use of BM chimeric mice, in which the BM of the recipient mice is depleted by lethal irradiation and replaced by transplantation of BM isolated from donor mice harboring the transgene of interest. Indeed, BM transplantation is widely used in clinical settings when treating leukemia patients. Richard and coworkers have taken a step forward and used direct LV injection into the mouse BM to achieve TLR2 expression in APP/PS1 transgenic/TLR2 knockout mice (APP/TLR2−/−) [66]. They showed that direct BM targeted LV-mediated TLR2 gene therapy restores the TLR2-deficiency-induced behavioral deficits in the treated mice.

Intracranial transplantations are notably invasive and, therefore, the administration of the cells via blood circulation would indeed be an outstanding option for cell therapy. If the cells are able to mediate their protective effects by direct phagocytosis, would it be possible to administer the phagocytic cells directly via blood circulation? This interesting aspect was studied in a series of experiments conducted by Lebson et al. in 2010 in a mouse model closely resembling the APPswe/PS1dE9 mice, the APP/PS1 mice [72]. The authors demonstrated that intravenously administered monocytes engraft the AD mouse brain. Moreover, monocytes genetically modified to overexpress an A*β* degrading enzyme, neprilysin, were able to clear preexisting A*β* from the brain parenchyma when the cells were given through jugular vein microport over a period of two months. Whether the amount of engrafted cells is sufficient to provide protection remains unanswered, since mere neprilysin overexpression in the peripheral muscle of triple transgenic AD mice has been shown to significantly reduce A*β* load in the brain parenchyma [39].

## 11. Concluding Remarks

The APPswe/PS1dE9 mice [16] display a variety of clinically relevant AD-like symptoms, including increased paranchymal A*β* load, inflammation, deficits in the cholinergic system, and cognitive impairment. Several studies aiming at therapeutic benefit have utilized these mice in the development of therapeutic approaches and as a model for human AD. The efficiency of gene therapy using viral vectors as gene transfer vehicles has been tested with several vector types and routes of administration with the ultimate goal of attenuating learning and memory deficits via depletion of A*β* and overexpression of protective genes. In addition, this mouse model has been utilized in cell-based therapy approaches aiming to remove toxic A*β* using BM stem cells, mesenchymal stem cells, and astrocytes. While the route of administration and potential harmful immune reactions remain the main hurdles, the results from the studies reviewed here, and further studies utilizing other AD mouse models imply that both gene therapy and cell-based therapy may be feasible therapeutic approaches for human AD.
